# Early Detection of Fusarium Basal Rot Infection in Onions and Shallots Based on VOC Profiles Analysis

**DOI:** 10.1021/acs.jafc.3c06569

**Published:** 2024-02-06

**Authors:** Malgorzata Wesoly, Emma Daulton, Sascha Jenkins, Sarah van Amsterdam, John Clarkson, James A. Covington

**Affiliations:** † Chair of Medical Biotechnology, Faculty of Chemistry, Warsaw University of Technology, Noakowskiego 3, Warsaw 00-664, Poland; ‡ School of Engineering, University of Warwick, Coventry Cv4 7AL, U.K.; § Warwick Crop Centre, School of Life Sciences, University of Warwick, Wellesbourne CV35 9EF, U.K.; ∥ AgResearch Ltd, Ruakura Research Centre, Hamilton 3214, New Zealand

**Keywords:** GC-IMS, ion-mobility spectrometry, plant disease detection, volatile organic compounds, Fusarium oxysporum f. sp. *cepae*, *Allium* species, pattern recognition, fungal plant disease

## Abstract

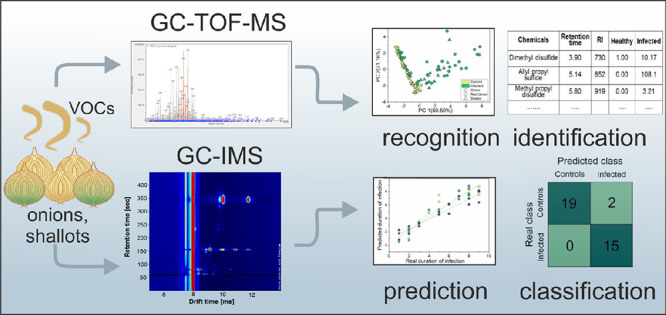

Gas chromatography ion-mobility spectrometry (GC-IMS) technology is drawing increasing attention due to its high sensitivity, low drift, and capability for the identification of compounds. The noninvasive detection of plant pests and pathogens is an application area well suited to this technology. In this work, we employed GC-IMS technology for early detection of Fusarium basal rot in brown onion, red onion, and shallot bulbs and for tracking disease progression during storage. The volatile profiles of the infected and healthy control bulbs were characterized using GC-IMS and gas chromatography-time-of-flight mass spectrometry (GC-TOF-MS). GC-IMS data combined with principal component analysis and supervised methods provided discrimination between infected and healthy control bulbs as early as 1 day after incubation with the pathogen, classification regarding the proportion of infected to healthy bulbs in a sample, and prediction of the infection’s duration with an average *R*^2^ = 0.92. Furthermore, GC-TOF-MS revealed several compounds, mostly sulfides and disulfides, that could be uniquely related to Fusarium basal rot infection.

## Introduction

Continued global population growth has led to increases in food demand. Minimizing food loss and waste is an essential strategy to meet such need without exploiting new agricultural areas.^1^ Pests, weeds, and pathogens play a key role in decreasing crop yield and quality.^2^ Globally, yield losses of five major crops (wheat, rice, maize, potato, soybean) caused by pests and pathogens have been estimated at 17–23%.^3^ Methods that detect and identify crop pathogens and pests at an early stage of infection/infestation are therefore of major importance in implementing appropriate management strategies to reduce losses.^4^

Onion (*Allium* cepa L.) is one of the most important horticultural crops cultivated worldwide, being grown in more than 140 countries.^5^ Over the last 10 years (2012–2022), world onion bulb production has increased by at least 30%, with current production being around 110.6 million tons, worth over $45 billion annually. When onion bulbs are harvested and thoroughly dried, they can be stored for up to 9 months in controlled-atmosphere storage facilities. This long-term storage characteristic, as well as durability for shipping, means that onion bulbs are widely traded worldwide.^6^

However, one of the main constraints to onion production and storage is the Fusarium basal rot caused by the soilborne fungal pathogen *Fusarium oxysporum* forma specializ (f.sp.) *cepae* (FOC). FOC infects the roots of onion plants, causing plant wilting and death in the growing crop, though most commonly results in basal rot of mature onion bulbs either in the field or store.^7^ The biggest losses for growers occur when bulbs are infected with FOC but with no apparent symptoms, with the disease expressed only in store. This can be exacerbated by poor drying and storage conditions that can then lead to loss of the entire stored crop.^8^

To minimize such pathogen-related crop losses in storage facilities, innovative methods that provide rapid, automated, and nondestructive crop disease detection are of particular interest. These include spectroscopic and imaging techniques, biosensors, and methods based on profiling of plant volatile organic compounds (VOCs) employing electronic nose systems, gas-chromatography mass-spectrometry (GC-MS), and gas-chromatography ion-mobility spectrometry (GC-IMS) among others. These tools represent a promising approach for monitoring and detecting plant pests and diseases, although they come with their own set of advantages and drawbacks.^9,10^

Volatile compounds play an important role in the interactions between plants and the surrounding environment. Their role in plant physiology has been extensively studied.^11,12^ VOCs emitted by plants are a rich source of information since they are strongly related to their health status and the stresses to which they have been subjected.^13^ Generally, plant volatiles are most often detected using GC-MS-based methods. Although these methods require expensive laboratory equipment and a trained user, they are state-of-the-art technologies, providing identification of a wide range of compounds, and often serve as a reference technique to novel tools that are still under development.^14^ Recently, Ficke et al.^15^ employed GC-MS to investigate VOCs associated with wheat fungal diseases and suggested that profiles are pathogen-specific and can be used for disease discrimination. Identification of plant pathogens based on GC-MS analysis of VOCs emitted by chilli plants,^16^ potatoes,^17^ and tomatoes^18^ have also been reported.

One of the promising strategies to detect and characterize VOC profiles of plants and agricultural products is IMS. Due to its low detection limits, IMS was initially used to detect dangerous substances, including chemical warfare agents, explosives, and drugs.^19^ Coupling GC with IMS results in improved separation efficiency as well as increased identification capabilities.^20^ GC-IMS technology is drawing increasing attention due to its high sensitivity, low drift, and capability for identification compounds. In recent years, much attention has been given to this technique in the fields of agricultural and food products. For instance, GC-IMS techniques have been employed for the detection and identification of VOCs from navel orange and pomelo leaves infected with bacteria,^21^ pickles,^22^ and brown rice infested by pests.^23^

This work presents a further study of the detection of Fusarium basal rot caused by FOC in onions and shallots based on volatile profiling. In our previous research, we demonstrated the capability of the PEN 3 electronic nose for long-term monitoring of Fusarium basal rot progression in onion and shallot bulbs stored under different temperatures.^24^ In this work, we focus on early detection of Fusarium basal rot, i.e., before visual symptoms occurred, predicting infection duration, as well as identification of detected VOCs. The volatile profiles of infected and healthy bulbs were characterized using GC-IMS and gas chromatography-time-of-flight mass spectrometry (GC-TOF-MS). To the best of our knowledge, this is the first report on the investigation of the feasibility of GC-IMS technology for the detection of Fusarium basal rot in brown onions, red onions, and shallot bulbs.

## Materials and Methods

### Sample Preparation

In the first experiment, 40 healthy brown onion and 20 shallot bulbs were used, obtained from Parrish Farms (FB Parrish & Sons Ltd., Bedfordshire, UK). Bulbs were subjected to a thorough examination to ensure health status; any with symptoms of Fusarium basal rot or other diseases including being soft, damaged, or having a “corky” basal plate indicative of an early FOC infection or insect infestation were discarded. Next, 20 healthy onion and 10 shallot bulbs were inoculated with an agar plug culture of FOC isolate FUS2 following a similar procedure described by Taylor et al.^25^ as detailed in our previous work.^24^ Briefly, a 2–3 mm slice of the basal plate of the bulbs was removed, and the bulbs were sprayed with 70% ethanol. Next an 8 mm plug of potato dextrose agar with the actively growing edge of a FOC isolate FUS2 colony was inverted and placed on the cut basal plate of each bulb. Control bulbs were prepared in the same manner, but no agar plug was placed on the basal plate. Following this inoculation, bulbs were placed in damp boxes in sealed plastic bags and incubated at 20 °C for 24 h. After this initial incubation, the healthy (noninoculated) control and inoculated bulbs were placed in the experimental system, which consisted of sealed 3 L plastic containers, with 3 mm gas inlet and outlet fittings added at both ends.

We then undertook a preliminary study of the feasibility of GC-IMS to differentiate between healthy and FOC-inoculated bulbs, track disease progression over time, and distinguish between samples stored under different conditions. For this task, inoculated and healthy control bulbs were stored for 5 weeks at 25 or 4 °C. In addition, a set of six samples with varying proportions of inoculated to healthy onion bulbs, i.e., 0, 20, 40, 60, 80, and 100% (5 bulbs in total) was also examined to evaluate the influence of the proportion of of diseased bulbs in a sample on its VOC profile.

In the second experiment, the capability of GC-IMS to detect Fusarium basal rot disease in different *Allium* species before visual symptoms occurred was investigated. Ten healthy brown onion bulbs, red onion bulbs, and shallot bulbs were used, obtained from a local market in Coventry, UK and checked for health status as before. Five healthy bulbs of each type were then inoculated with FOC isolate FUS2 as in the first experiment. After initial incubation at 20 °C for 24 h, five bulbs of each type were placed in sealed 3 L plastic containers. A set of 12 samples, each consisting of five healthy or five FOC-inoculated bulbs of each type, were stored for 9 days at 25 °C. Throughout both experiments, high humidity was maintained in the containers to ensure ideal conditions for FOC infection to develop. At the end of each experiment (i.e., after 31 and 9 days of storage, first and second experiment, respectively), the bulbs were cut open to confirm symptoms of FOC infection and healthy status of control bulbs. No visible symptoms of the FOC infection were observed in the noninoculated bulbs.

### GC-IMS Analysis

The GC-IMS used in this study was a G.A.S. GC-IMS (G.A.S., Dortmund, Germany). The instrument is formed from a gas chromatography (GC) column (FS-SE-54-CB-1, 30 m × 0.44 mm (OD) × 0.32 mm (ID), CS Chromatographie Service GmbH, Langerwehe, Germany) followed by a drift tube ion mobility spectrometer. Pure nitrogen was used as a buffer gas, which flows in the opposite direction to the ions, resulting in ion/nitrogen collisions. The GC-IMS was operated using the following settings: GC flow rate 20 mL/min for 4 min, drift tube flow rate 150 mL/min for 6 min, temperature 45 °C (IMS), temperature 80 °C (GC column), and temperature 70 °C (sample port). The total run time per sample was 10 min. The valve responsible for allowing the sample to flow into the GC was opened for a total of 6 s at a rate of 20 mL/min, allowing 2 mL of the sample headspace to be used for analysis. The plastic container containing bulb sample was connected with the GC-IMS inlet, and the headspace above the bulbs was injected into the instrument to identify and measure VOCs (Figure S1). In the first experiment, the GC-IMS measurements were carried out twice a week, whereas during the second experiment, measurements were carried out daily.

### GC-TOF-MS Analysis

A Markes GC-TOF-MS consisting of a TRACE 1300 GC (Thermo Fisher Scientific, Loughborough, UK) and Bench TOF-HD TOF-MS (Markes Intl., Llantrisant, UK) was used for the VOC analysis of the bulb samples. The system also has a high-throughput autosampler and thermal desorption unit, ULTRA-xr and UNITY-xr, respectively (Markes International, UK).

To collect volatiles, a thermal desorption sorbent tube (C2-AXXX-5149, Markes Intl., Llantrisant, UK) was placed in each sample container for 24 h after which they were analyzed. The following settings were used for the ULTRA-xr: stand-by split set to 150 °C and GC run time 25 min with a programmed temperature ramp from 40 to 280 °C at 20 °C/min. The ionization was set to −70 V. There was a 1 min prepurge for each sample followed by desorption for 10 min at 250 °C and trap purge for 1 min. The trap was then cooled to 30 °C followed by a 3 min purge at a temperature of 300 °C. Both the transfer line and ion source were heated to 250 °C. Once the data was obtained, the samples were processed using TOF-DS (Markes International, UK), a dynamic background compensation was applied, and peaks were integrated and deconvoluted. Integration settings were as follows: global height reject 10,000, global width reject 0.001, baseline threshold 4, and global area reject 10,000. The sample peaks were identified using NIST 2020, with forward and reverse matching set to 450 and verified with standards by comparing calculated retention indices (RI). GC-TOF-MS analysis was run 11 and 8 days after bulb inoculation, in the first and second experiment, respectively.

### Data Analysis

**Figure 1:**
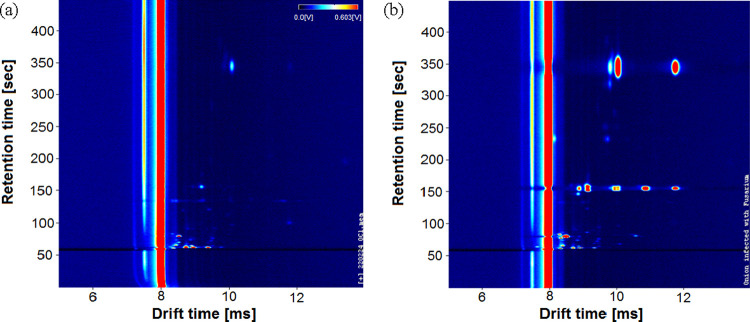
Typical output plots from GC-IMS instrument: (a) healthy onion samples; (b) FOC-inoculated onion samples 9 days after inoculation.

For GC-IMS data analysis, IMS spectra were processed using the G.A.S VOCal (v0.1.3, G.A.S., Dortmund, Germany) software. A typical output from the GC-IMS for a healthy control and an infected sample is shown in Figure 1. In this figure, the red and light blue areas indicate that the instrument detects chemicals, whereas the background is displayed as a dark blue area, where there are no chemicals present. The intensity of the peak (with red being the highest intensity) represents the number of ions and thus related to the chemical abundance. The vertical red line in the figure is the default output of the instrument when no chemicals are present. Figure 1 also shows that extensive chemical information was collected from the samples.

The GC-IMS creates very high dimensional data sets, totalling around 11 million data points per sample. Therefore, the data analysis involved a preprocessing stage to extract only the information necessary to build and train diagnostic models. Using the VOCal software, 23 and 26 areas (the first and second experiment, respectively) were identified by visual inspection, and the maximum peak height within selected areas was extracted for all the samples at the same locations and then were subjected to chemometric analysis.

To explore the GC-IMS data and reduce its dimensionality without loss of information, principal component analysis (PCA) was applied.^26^ Next, several supervised methods specifically, linear discriminant analysis (LDA), partial least squares—discriminant analysis (PLS-DA), support vector machine—discriminant analysis (SVM-DA), and K-Nearest Neighbors (KNN) were employed to build classification models. Finally, a PLS regression model was built to predict the duration of infection, i.e., the number of days after inoculation. Evaluation of the classifiers’ performance was based on accuracy, recall, and F1 score.^27^ In the case of multiclass classification, these parameters were averaged from parameters calculated per each class. Regression PLS models were examined based on the determination coefficient (*R*^2^) calculated between measured and predicted number of days after inoculation. Data processing was carried out with MATLAB R2020a (The Math-Works Inc., Natick, MA, USA), Solo (eigenvector Research Inc., Manson, WA, USA), and Origin 2021b (OriginLab Corporation, Northampton, MA, USA) software.

For GC-TOF-MS, the chemicals and the abundance of the chemicals were determined using the TOF-DS software. A background correction was applied, and the chromatogram was integrated, and the peaks were identified using the NIST list. Around 500 peaks were detected for each sample. The data obtained from GC-TOF-MS were converted into text files of chemical lists and abundances. The chemical compounds that were in less than half of the studied set of samples were excluded from the analysis. Next, the chemical compounds and their discriminative power were evaluated based on *t*-student test; the compounds with *p*-value <0.05 were considered as significant. The RI of each compound was calculated based on the relative retention times of a series of *n*-alkanes (C7–C20, Sigma-Aldrich, St. Louis, MO, USA). During the GC-TOF-MS analysis, it was observed that few compounds with the shortest retention times eluted before the first hydrocarbon in the *n*-alkanes mixture precluding the determination of RI for these analytes. The semiquantification of significant compounds was performed using their absolute peak heights and calculating relative abundance with respect to healthy control samples stored at 25 °C.

## Results and Discussion

### GC-IMS Data Analysis

#### Principal Component Analysis

**Figure 2:**
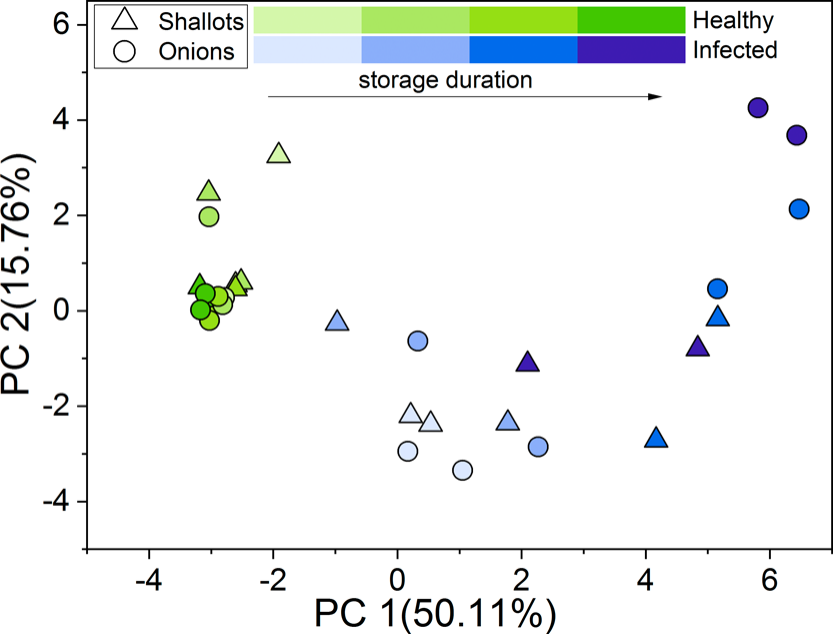
PCA scores plot of volatile profiles’ patterns of samples consisting of FOC-infected and healthy control onion and shallot bulbs stored at 25 °C.

Patterns of the signals’ intensities from the selected 23 areas of GC-IMS spectra representing VOC profiles of samples examined in the first experiment (preliminary studies) were analyzed using PCA. Prior processing variables were auto-scaled. The PCA score plot of VOC profiles of samples containing FOC-infected or healthy onion or shallot bulbs is shown in Figure 2. Each point represents a different duration of infection from 1 to 4 weeks. Clear distinctions between these two groups of samples can be observed. One cluster characterized by the lowest PC1 values forms points corresponding to healthy control bulbs, while the second one is formed by the points representing infected bulbs, even though the points are spread across the plot. Therefore, FOC-infected onion and shallot bulbs stored at 25 °C have different volatile profiles compared to healthy control bulbs. It is worth noting that points corresponding to the first week postinfection can be clearly distinguished from the points representing healthy samples. Moreover, the progress in FOC infection can be associated with the points’ position on the plot; i.e., as the infection progresses over time, the corresponding sample PCA location moves position along PC1. Finally, the loading plot, presented in Figure S2, revealed that all 23 of the selected 23 areas contributed significantly to the differences among samples.

Next, the differences in the VOC profiles related to varying proportions of FOC-infected and healthy onion bulbs were evaluated using PCA. The score plots of the volatile profiles’ patterns of samples consisting of varying proportions of FOC-infected and healthy onion bulbs are shown in Figure S3. Each point represents a different duration of infection. To clarify the visualization of the trend of changes in volatile profiles, PCA was applied to processed data from three groups of samples (0, 40, and 100%), and the resulting score plot is shown in Figure S3b.

Points corresponding to healthy bulbs are located close to each other and formed one cluster, characterized by the lowest values of PC1 and PC2. Close to the points representing healthy control samples are points corresponding to the sample containing 20% FOC-infected bulbs at an early stage of infection. The points corresponding to the greatest proportion of infected bulbs in the sample are characterized by the highest PC1 value. This indicates that the position of the point on the plot can be related to the proportion of the infected bulbs in the sample. Also, the greater durations of infection (i.e., storage time) are reflected by points characterized by higher PC1 and PC2 location values.

PCA was also employed to investigate the influence of the storage temperature on the volatile profiles of the samples. The higher storage temperature was shown to accelerate FOC infection. The score plot of the volatile profiles’ patterns of the infected onion and shallot bulbs stored at different temperatures is shown in Figure S4. Clear distinction between points corresponding to FOC-infected samples stored at different temperatures can be observed. Points representing infected bulbs stored at 25 °C are spread across the PCA plot; thus, significant changes in the volatile profiles related to the progression of infection were found. This confirms that 25 °C is a much more favorable temperature for the development of Fusarium basal rot compared with 4 °C. Also, as was observed by Liu et al.,^28^ higher temperature of storage increases the concentration of VOCs emitted by healthy onion bulbs. This result was in line with our findings that VOCs emission rates are higher in the headspace of both healthy control and infected bulbs stored at 25 °C in comparison to 4 °C.

**Figure 3:**
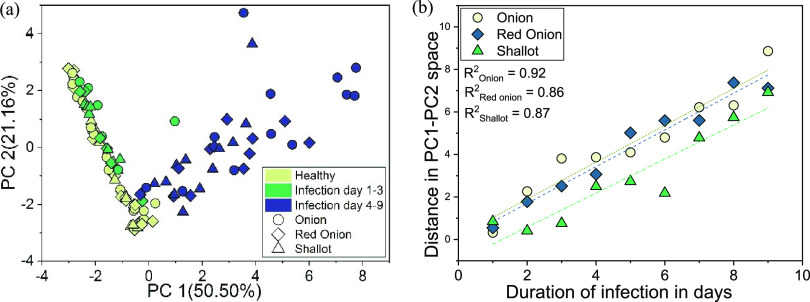
(a) PCA scores plots of volatile profiles’ patterns of samples consisting of FOC-infected and healthy brown onion, red onion, and shallot bulbs. (b) Correlation between duration of infection and Euclidean distance in PC1–PC2 space.

The above result indicated that GC-IMS data can be used to discriminate between healthy and diseased bulbs and assess the progress of FOC infection over time. We then undertook further studies (second experiment), using GC-IMS, as a tool for the early detection of Fusarium basal rot. First, PCA was employed to 26 areas of GC-IMS spectra, however, based on visual examination of the resulting score and loading plots (data not shown), 14 features were extracted and the resulting PCA score plot and loading plot are shown in Figures 3a and S5, respectively. Six samples were prepared in duplicate; therefore, each of the two points represents a duration of infection from 1 to 9 days. Two groups of points can be easily discerned on the plot, representing healthy control and FOC-infected bulbs. However, points corresponding to the infected bulbs at an early stage of infection also formed one cluster, with points overlapping healthy control samples. This suggests that volatile profiles of the infected bulbs 1–3 days after incubation, are similar to those of healthy control samples (with minimal variance). With increasing duration of infection, corresponding points on the plot were characterized by increased PC1 and PC2 location values and with increased distance to points corresponding to healthy control samples. The calculated Euclidean distances between points representing healthy control and FOC-infected bulbs of the same type over time provided a quantitative measure of these differences. A strong linear correlation was found, with *R*^2^ values ranging from 0.86 to 0.92 (Figure 3b). The longer the infection duration, the greater the distance between points on the PCA plot. It was also found that all of the 14 selected areas of IMS spectra contributed significantly to the differences among samples, indicating that a wide range of biomarkers are modulated in the disease state (Figure S5).

Wang et al.,^29^ who investigated fungal infection in walnuts using GC-IMS, also reported discrimination between diseased samples and healthy controls based on PCA. This is therefore further evidence that GC-IMS technology provides relevant data for the detection of fungal infection during storage of different crops.

### Classification of the Samples

PCA results demonstrated that the volatile profiles of the samples collected using GC-IMS are a rich source of relevant information about the progression of FOC infection in onion and shallot bulbs. Moreover, volatile profiles of the samples containing varying proportions of infected bulbs differed from each other as well as over time. Therefore, classification models were built using four commonly used supervised methods, namely, LDA, SVM-DA, KNN, and PLS-DA to

1. Differentiate between healthy control and FOC-infected shallot and onion bulbs during long-term storage; i.e., samples consisting of 0 and 100% of infected bulbs—2 class classification;
2. Assign samples into one of three groups relating to the proportion of infected onion bulbs in a sample, i.e., healthy control (0% infected bulbs), mildly diseased (20–40% infected bulbs), and severely diseased (60–100% of infected bulbs)—3 class classification;
3. Differentiate between healthy control and FOC-infected brown onion, red onion, and shallot bulbs at early stage of infection—2 class classification.

A 10-fold cross-validation was used to validate the classification ability of the models built in the first experiment. Prior to the data processing, variables were auto-scaled. The reported data here are for the cross-validated test data set. In the second experiment, a data set was split between the train and test set (2:1), and the reported data are for the external test set, which was not used during the training model. The accuracy, recall, and F1 score of the models are shown in Table 1. The confusion matrices representing the results of the classification based on volatiles collected in the second experiment are shown in Figure S6.

**Table 1:** Accuracy, Recall, and F1 Score of the Classification Models Developed Based on LDA, SVM-DA, KNN, and PLS-DA Algorithms

		algorithm	accuracy	recall	F1 score
preliminary studies	2 class classification	LDA	100.0%	1.0	1.0
		SVM-DA	100.0%	1.0	1.0
		KNN	100.0%	1.0	1.0
		PLS-DA	100.0%	1.0	1.0
	3 class classification	LDA	87.5%	0.86	0.87
		SVM-DA	89.6%	0.89	0.90
		KNN	85.4%	0.87	0.87
		PLS-DA	81.3%	0.83	0.89
early disease detection	2 class classification	PLS-DA	94.4%	0.95	0.94
		SVM-DA	91.7%	0.90	0.91
		KNN	86.1%	0.88	0.86
		LDA	91.7%	0.90	0.93

All developed models perfectly distinguished healthy control bulbs from FOC-infected bulbs from the first week of infection, with no misclassified samples. Multiclass classification resulted in a slightly worse performance of models, having accuracy ranging from 81.3 to 89.6%, PLS-DA with seven latent variables, and SVM-DA, respectively. SVM-DA which employed the radial basis function was found to provide the best performance with almost 90% accuracy, 0.89 recall, and 0.9 F1 score. Therefore, volatile profiles were found to be related to both whether the bulbs were infected and the proportion of infected bulbs in a sample.

The results of these preliminary studies demonstrate that supervised methods can be successfully applied to discriminate between healthy control and FOC-infected bulbs and that GC-IMS is potentially a suitable tool for early detection of Fusarium basal rot. In this case, all models successfully classified most of the samples with accuracy ranging from 86.1 to 94.4%. PLS-DA model based on nine latent variables showed the best performance having the highest accuracy, recall, and F1 score, 94.4%, 0.95, and 0.94, respectively. Also, SVM-DA and LDA models correctly classified more than 90% of the samples, having recall and F1 scores of more than 0.90. These findings correspond well with the work of Wang et al.^8^ where the extent of Fusarium basal rot in onion bulbs detected by real-time polymerase chain reaction (PCR) was related to the total volatile emission rate and the amount of pathogen DNA. Sinha et al.^30^ employed Field Asymmetric Ion Mobility Spectrometry (FAIMS) for real-time detection of soft rot bacterial pathogens in stored potatoes and onions. In this case, Naïve Bayes and LDA classifiers were applied to process the FAIMS data. The reported classification accuracies for detecting the bacterial pathogen *Burkholderia cepacia* causing sour skin in onions were around 70% for the 0 and 1 day after inoculation and significantly higher (up to 100%) between 4 and 16 days after inoculation. The results presented here showed that a PLS-DA model could recognize an infected sample as early as 1 day after incubation with the pathogen with 94% accuracy, though this may also be related to the inoculation process. It is challenging to guarantee 100% detection of disease, but based on the results, a sufficient time of incubation with the pathogen is less than 7 days.

In our previous work,^24^ we demonstrated that the PEN 3 electronic nose was effective for detection and monitoring of Fusarium basal rot disease development in onion and shallot bulbs artificially infected with FOC. In comparison, the GC-IMS, from this study, was found to be slightly better at distinguishing between healthy control and FOC-infected onion and shallot bulbs (100% accuracy, compared with 97%, and 1.0 to 0.94 recall). PCA results also indicated that GC-IMS data provide clearer differentiation between infected and healthy control bulbs than the electronic nose data. However, both technologies showed similar classification abilities considering multiclass classification, i.e., distinguishing between healthy control, mild, and severely diseased samples.

### Prediction of the Duration of FOC Infection

The next step was to investigate the capability of GC-IMS to track the progress of FOC infection and detect Fusarium basal rot early using a quantitative approach. The peak heights of the 26 descriptive areas from GC-IMS data were processed using PLS, and the aim of this analysis was to predict the duration of FOC infection in days (postincubation). The data set was randomly split into train and test set (2:1) three times, and therefore, three independent PLS regression models were developed. To evaluate the models’ predictive power, external data sets were used. Prior processing variables were auto-scaled. Correlation between the measured duration of infection and those predicted by three independent PLS models is shown in Figure S7. The reported results are for the test data sets. A strong linear correlation was found between real and predicted values of the duration of FOC infection for all three PLS models regardless of the bulb type. Regression coefficient (*R*^2^) ranged from 0.88 to 0.94, and average was 0.92. This result demonstrates that, based on GC-IMS data, a PLS model can successfully track Fusarium basal rot disease progression over time even after 1 day of incubation with the pathogen.

### Identification of VOCs Using GC-MS Analysis

A total of 37 significant volatile compounds were identified in the headspace of containers across all the samples analyzed in the first experiment, i.e., those with the varying proportions of FOC-infected onion bulbs and healthy control bulbs, and those with FOC-infected onion and shallot bulbs stored at 4 or 25 °C. Chemical compounds were identified using TOF-MS software but were not calibrated for concentration. However, the identification of VOCs was confirmed with standards based on the comparison of individual Kovats indices. A few compounds were found to show different RIs than the standards, but this may be related to the midpolar column that was used in our study. Volatiles detected in the samples and their relative abundance are presented in Tables 2 and S1. Among them, 8 VOCs were identified in previous research^8,31,32^ as related to Fusarium infection in onion or apple; these included 2,2-bis(methylthio)propane, 3,4-dimethyl thiophene, allyl propyl sulfide, dimethyl disulfide, isopropyl disulfide, methyl propyl sulfide, methyl propyl disulfide, and styrene. These compounds were found to be emitted by both healthy control and FOC-infected bulbs but with significantly different abundance. The challenge in the detection of fungal infections in onions and shallots lies in the abundance of related sulfur compounds in healthy samples, such as methyl propyl disulfide.^33^ Prithiviraj et al.^31^ detected 42 VOCs in high abundance from onion bulbs infected with the bacterial pathogen *Erwinia carotovora*, or the fungal pathogens *Botrytis cinerea* or FOC. 10 identified VOCs were unique to these pathogens, but FOC produced two unique metabolites 1-oxa-4,6-diazacyclooctane-5-thione and 4-mercapto-3-(methylthio)-*ç*-(thio-lactone)-crotonic acid. However, these two compounds were not found in this study.

**Table 2:** VOCs Identified Using GC-TOF-MS in the Preliminary Studies and Their Calculated RI^a^

chemicals	retention time (min)	RI
cyclohexane	2.86	n.d.
methyl propyl sulfide	3.50	n.d.
3-ethyl-pentane	3.86	727
dimethyl disulfide	3.92	733
3,4-dimethylthiophene	5.16	853
allyl propyl sulfide	5.16	854
propyl sulfide	5.31	868
styrene	5.42	880
2,7-dimethyl octane	5.49	886
octamethyl-cyclotetrasiloxane	5.64	901
methyl allyl disulfide	5.71	908
4-methyl nonane	5.80	918
methyl propyl disulfide	5.83	922
2-pentyl furan	6.22	962
1-methyl-4-(1-methylethylidene)-cyclohexene	6.48	990
2,3,6,7-tetramethyl octane	6.63	1006
β-phellandrene	6.65	1008
2,2-bis(methylthio)propane	6.76	1021
[(4-hexylbenzene-1,3-diyl)bis(oxy)]bis(trimethylsilane)	7.27	1079
5-methyl-5-propyl nonane	7.28	1080
1-methylethyl 2-propenyl disulfide	7.33	1085
(*E*)-1-propenyl allyl disulfide	7.29	1092
isopropyl disulfide	7.44	1098
2-methyl undecane	7.57	1114
2,6,11-trimethyl dodecane	7.66	1125
hexadecane	7.90	1153
2,6-dimethyl undecane	7.97	1162
4-methyl dodecane	8.04	1171
4-ethyl phenol	8.39	1215
pentadecane	8.48	1226
hexylbenzene	8.57	1238
4-methyltridecane	9.06	1302
2-methyl tridecane	9.10	1307
dipropyl trisulfide	9.23	1325
3-isopropoxy-1,1,1,7,7,7-hexamethyl-3,5,5-tris(trimethylsiloxy)tetrasiloxane	9.85	1411
2-tridecanone	10.32	1480
1,14-tetradecanediol	11.07	1595

a n.d.: no data.

In other work, Wang et al.^8^ identified ethanol, 1-propanethiol, methyl propyl sulfide, dimethyl disulfide, styrene, methyl propyl disulfide, and 2,2-bis(methylthio)propane as compounds associated with FOC-infected onions. These compounds had high average emission rates from FOC-infected onions, and those correlated well with the FOC DNA ratio. However, they were found in the headspaces of both infected and healthy control bulbs, except styrene. VOCs including propene, methanethiol, (*E*)-1,3-pentadiene, C_5_H_8_, 1-propanol, methyl isopropyl sulfide, 3-methyl-1-butanol, and 2-heptanone were found to be emitted only by FOC-infected bulbs, although emission rates were low.

In this work, 10 VOCs, namely, methyl propyl sulfide, methyl allyl disulfide, 1-methyl-4-(1-methylethylidene)-cyclohexene, β-phellandrene, 2,2-bis(methylthio)propane, 5-methyl-5-propyl nonane, 1-methylethyl 2-propenyl disulfide, (*E*)-1-propenyl allyl disulfide, dipropyl trisulfide, and 2-tridecanone were found in high abundance in the headspace of FOC-infected bulbs and none or almost none in the headspace of the healthy control bulbs (Table S1). In our experiments, these compounds were uniquely associated with Fusarium basal rot in onion bulbs. Also, a strong linear correlation with *R*^2^ ranging from 0.84 to 0.90 was found between the proportion of FOC-infected bulbs in a sample and the abundance of β-phellandrene, 2,2-bis(methylthio)propane, 1-methylethyl 2-propenyl disulfide, dipropyl trisulfide, and allyl propyl sulfide. Therefore, these compounds are the most promising volatile biomarkers of FOC infection in onions and shallots.

Fewer VOCs and with lower abundance were detected in the samples stored at 4 °C. Only styrene was presented in high abundance, which we believe is associated with the containers used in the experiments. Negligible differences in the abundance of sulfides between healthy control and infected samples stored at 4 °C were found again suggesting that the lower temperature of storage significantly inhibits the development of fungal FOC infection as found in previous studies.^24^

Next, VOCs collected from the internal space of the containers with healthy control and infected brown onion, red onion, and shallot bulbs during the second experiment were analyzed using GC-TOF-MS. A total of 49 significant volatile compounds were found and identified by using TOF-MS software. A list of the VOCs and their relative abundances of compounds detected in both experiments is shown in Table 3. The remaining VOCs identified in the headspace of healthy control bulbs and FOC-infected bulbs in early detection studies are listed in Table S2. Nine compounds were identified in the headspace of the healthy control and FOC-infected bulbs in both experiments. Again, sulfides and disulfides, β-phellandrene, and 2,2-bis(methylthio) propane were found in relatively high abundance in the headspace of infected bulbs. This trend was also evident for pentane, allyl methyl sulfide, 2,4-dimethyl thiophene, 1,2-dithiolane, 2-undecanone, and 2-hexyl-5-methyl-3(2*H*)-furanone. Therefore, these compounds are potentially related to FOC infection in onions and shallots. Several of these VOCs including 2,2-bis(methylthio)propane and methyl propyl sulfide were suggested as a potential biomarkers of Fusarium basal rot in other studies.^8,34^ A lower relative abundance of potential biomarkers, such as methyl propyl sulfide, β-phellandrene, and 2,2-bis(methylthio)propane were detected in infected red onion bulbs in comparison with brown onions and shallot bulbs.

**Table 3:** List of VOCs and Their Relative Abundance Detected in Both Experiments^a^

chemicals/relative abundance	retention time (min)	RI	healthy control samples			FOC-infected samples		
			brown onion	red onion	shallot	brown onion	red onion	shallot
methyl propyl sulfide	3.48	n.d.	0.00	1.00	1.00	∞	4.68	63.59
dimethyl disulfide	3.90	730	1.00	1.00	1.00	3.21	6.73	108.09
allyl propyl sulfide	5.14	852	0.00	0.00	0.00	∞	∞	∞
methyl propyl disulfide	5.80	919	1.00	1.00	1.00	3.34	2.97	20.18
octamethyl-cyclotetrasiloxane	5.88	926	1.00	1.00	1.00	0.00	0.78	0.32
2-pentyl furan	6.19	960	1.00	1.00	1.00	2.38	1.17	2.38
β-phellandrene	6.62	1005	0.00	1.00	0.00	∞	20.51	∞
2,2-bis(methylthio)propane	6.73	1018	0.00	1.00	0.00	∞	3.09	∞
4-methyl dodecane	8.01	1167	1.00	1.00	1.00	1.50	8.22	0.66

a n.d.—no data. ∞—lack of compound in volatile profile of healthy control sample and simultaneously significant abundance in volatile profile of infected sample.

Differences in the compounds identified in the first and second experiment hinder the interpretation of the results. Those differences can be related to several reasons, including the inoculation procedure, rate of disease progression, individual volatile profile of the crop, and statistical significance of each compound. Using GC-TOF-MS around 500 compounds were found in the volatile profile of each sample. We excluded most of them, based on *t*-student test to focus on those that were significant, but this could have resulted in variations of the volatile profiles across replications. Also, as observed by Prithiviraj et al.,^31^ composition of the volatile profiles of healthy and infected bulbs could vary over replications of the measurements.

This study has several limitations. First, the sample size was small, although we did repeat measurements, and small sample sets are less prone to inconsistent infection. Furthermore, we did not confirm the chemical compounds using authentic gas reference compounds or were able to quantify the concentrations. However, the identified chemicals did correspond to previously published works, suggesting that our chemical identification is plausible. Finally, our experiments were undertaken in laboratory conditions rather than in commercial onion stores. We did, however, replicate store conditions by investigating different temperatures.

GC-IMS technology offers high sensitivity, short, cost-effective analysis, and results that are easier to interpret than those from the classic GC-MS method. In this study, it was used as a laboratory method; however, versions of these units are used as a point of analysis tool for many different industrial processes (such as beer and milk products). The practical implementation of GC-IMS technology presents significant challenges, primarily driven by the difficulty in implementing it in a selection line in-store facilities. However, the dynamic development of this technology shows promise as a screening method for the detection of crop disease. Further work is needed to understand its use in more complex odor environments.

Our findings proved the feasibility of GC-IMS to detect Fusarium basal rot infection even before the occurrence of visible symptoms in onion and shallot bulbs and to track disease progression during storage. A noninvasive detection method for fungal postharvest disease is of particular interest for managing disease in storage facilities. In further work, we aim to carry out studies in commercial storage conditions as well as expand testing with GC-IMS to differentiate between different postharvest pathogens in the stored crop.

## Supporting Information Available

Additional experimental results include: PCA results, confusion matrices of the classification results, photographs of the experimental setup, and a list of VOC detected in the second experiment (PDF)

## Abbreviations

FAIMS: field asymmetric ion mobility spectrometry
FOC: *Fusarium oxysporum* forma specializ *cepae*
GC-IMS: gas-chromatography ion-mobility spectrometry
GC-MS: gas-chromatography mass-spectrometry
GC-TOF-MS: gas chromatography–time-of-flight mass spectrometry
KNN: K-nearest neighbors
LDA: linear discriminant analysis
PCA: principal component analysis
PCR: polymerase chain reaction
PLS-DA: partial least squares-discriminant analysis
RI: retention indices
SVM-DA: support vector machine-discriminant analysis
VOCs: volatile organic compounds
