# Supplementary material for: Early Detection of Fusarium Basal Rot Infection in Onions and Shallots Based on VOC Profiles Analysis
Source: J Agric Food Chem. 2024 Feb 6;72(7):3664–72. doi: 10.1021/acs.jafc.3c06569 (PMC10885136; doi:10.1021/acs.jafc.3c06569)
Supplement: Supplementary file 1 — jf3c06569_si_001.pdf [file jf3c06569_si_001.pdf]

Early detection of fusarium basal rot infection in onions and shallots based on VOC profiles analysis

Malgorzata Wesoly[1,*], Emma Daulton[2], Sascha Jenkins[3], Sarah van Amsterdam[4], John Clarkson[3], James A. Covington[2,*]

[1]Chair of Medical Biotechnology, Faculty of Chemistry, Warsaw University of Technology, Noakowskiego 3, Warsaw, 00-664, Poland; malgorzata.wesoly@pw.edu.pl (M.W.)
[2]School of Engineering, University of Warwick, Coventry, Cv4 7AL, UK; e.daulton@warwick.ac.uk (E.D.), j.a.covington@warwick.ac.uk (J.A.C.)
[3]Warwick Crop Centre, School of Life Sciences, University of Warwick, Wellesbourne CV35 9EF, UK; sascha.jenkins.1@warwick.ac.uk (S.J.); john.clarkson@warwick.ac.uk (J.P.C.)
[4]AgResearch Ltd, Ruakura Research Centre, Hamilton 3214, New Zeland; Sarah.vanAmsterdam@agresearch.co.nz (S.v.A.);

*Correspondence: malgorzata.wesoly@pw.edu.pl (M.W.), j.a.covington@warwick.ac.uk (J.A.C.)

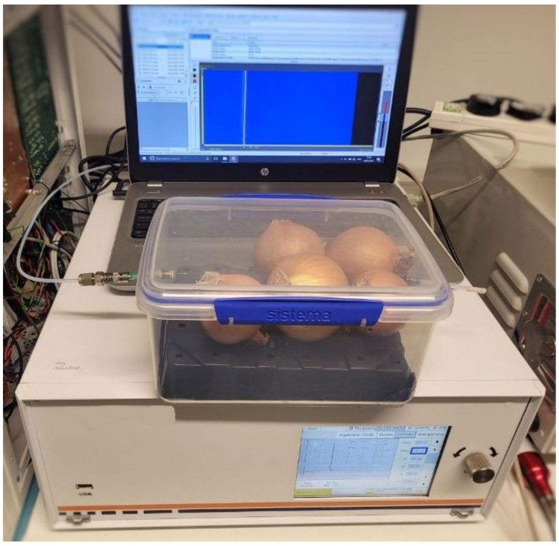

Figure S1. Laboratory setup for the measurements of volatiles released by bulbs using GC-IMS.

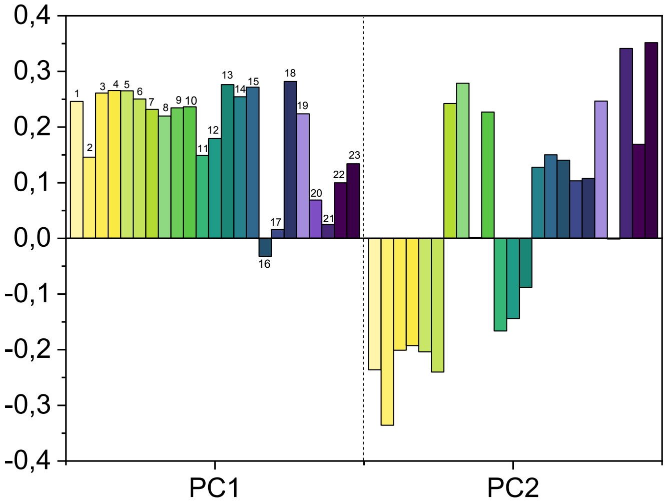

Figure S2. PCA loading plot of 23 areas selected from IMS spectra representing VOC profiles of samples examined in the first experiment.

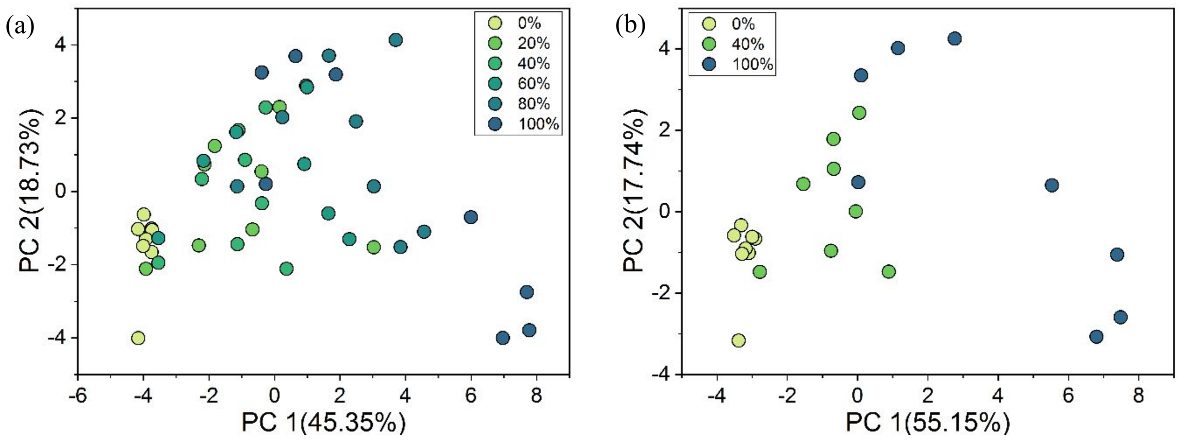

Figure S3. PCA score plots of the volatile profiles' patterns of samples consisting of infected and healthy onion bulbs at varying proportions: (**a**) six groups of samples, (**b**) three groups of samples.

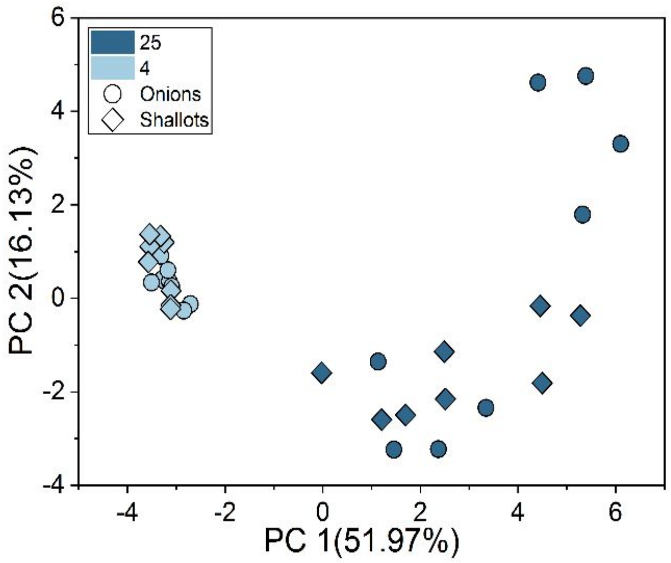

Figure S4. PCA score plot of the volatile profiles' patterns of the infected onion and shallot bulbs stored at different temperatures.

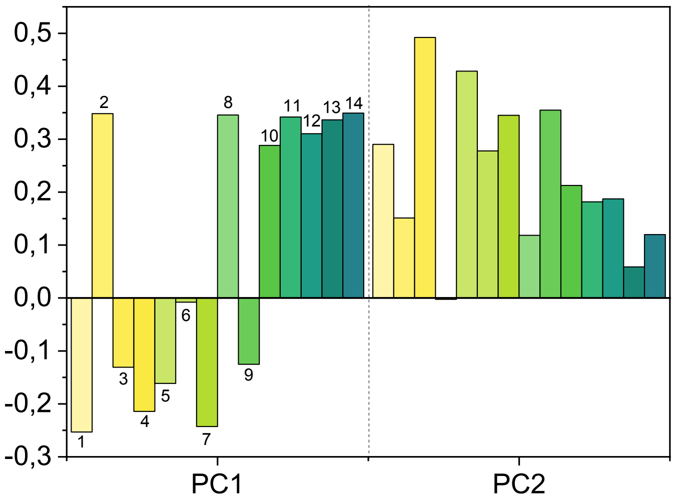

Figure S5. PCA loading plot of 14 areas selected from GC-IMS spectra representing VOC profiles of samples studied in the second experiment.

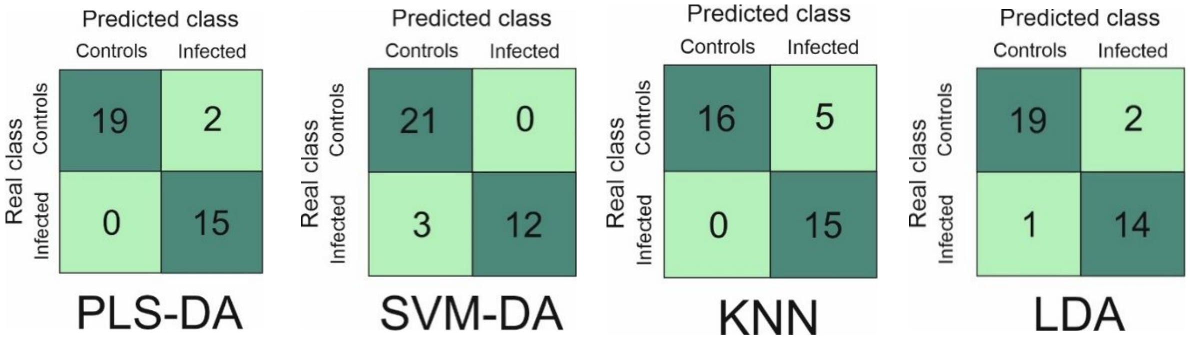

Figure S6. The confusion matrices of the classification results for the testing dataset.

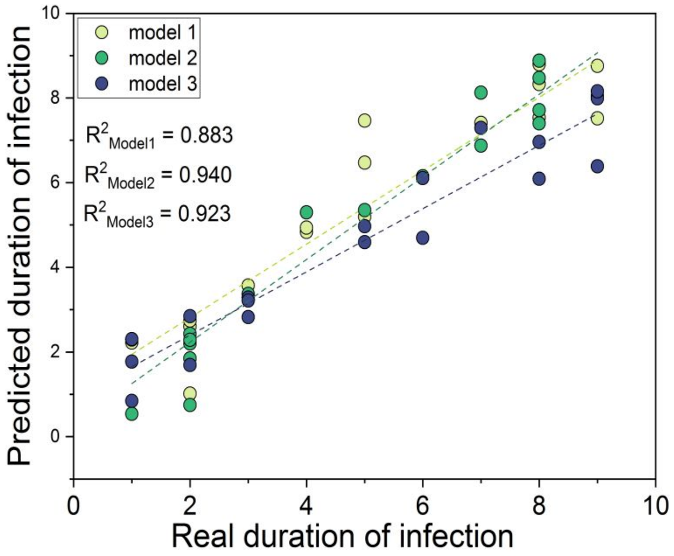

Figure S7. Correlation between real durations of FOC infection and those predicted by three PLS models based on GC-IMS data.

Table S1. The VOCs and their relative abundance detected inside the containers with healthy control and FOC-infected onion and shallot bulbs in the preliminary studies.

**Chemicals/ Relative abundance**	**Onion bulbs stored at 25°C**						**Onion bulbs stored at 4°C**		**Shallot bulbs stored at 25°C**		**Shallot bulbs stored at 4°C**	
	Proportion of infected to healthy bulbs in a sample (0%=healthy bulbs only)											
	0%	20%	40%	60%	80%	100%	0%	100%	0%	100%	0%	100%
Cyclohexane	1.00	6.29	10.64	2.70	0.57	10.65	3.83	29.41	1.00	0.10	2.42	3.16
Methyl propyl sulfide	1.00	35.32	47.68	18.86	24.22	55.04	0.24	0.00	1.00	19.72	0.00	0.00
3-ethyl-pentane	1.00	1.55	0.00	0.06	0.00	0.02	0.00	0.00	1.00	0.22	0.00	0.10
Dimethyl disulfide	1.00	2.24	3.36	1.78	4.04	3.15	0.02	0.01	1.00	2.45	0.01	0.23
3,4-dimethylthiophene	1.00	2.31	5.31	4.06	10.67	6.86	0.00	0.00	1.00	0.45	0.97	0.00
Allyl propyl sulfide	1.00	1.70	2.56	1.85	4.29	5.47	0.00	0.00	0.00	∞	∞	0.00
Propyl sulfide	1.00	4.06	2.53	1.91	2.01	2.12	0.04	0.00	0.00	∞	∞	0.00
Styrene	1.00	3.19	1.80	1.35	1.38	1.95	0.65	2.01	1.00	1.23	0.68	2.89
2,7-dimethyl octane	1.00	2.62	2.63	1.82	1.28	1.96	0.11	0.00	1.00	0.52	0.00	0.00
Octamethylcyclotetrasiloxane	1.00	1.49	0.23	0.77	0.56	0.00	1.25	1.28	1.00	0.00	0.55	1.90
methyl allyl disulfide	1.00	6.40	12.65	5.32	19.66	16.39	0.00	0.00	1.00	22.09	0.00	0.00
4-methyl nonane	1.00	0.87	1.07	0.22	0.47	0.89	0.13	0.08	1.00	0.00	0.11	0.08
Methyl propyl disulfide	1.00	1.91	1.98	2.12	1.89	2.12	0.07	0.09	1.00	2.86	0.09	0.23
2-pentylfuran	1.00	1.14	1.29	1.67	3.15	1.55	0.18	0.08	1.00	1.11	0.20	0.10
1-methyl-4-(1-methylethylidene)-cyclohexene	1.00	6.05	0.70	5.87	14.27	15.63	0.11	0.00	0.00	∞	∞	∞
2,3,6,7-tetramethyloctane	1.00	0.00	0.00	0.00	0.93	0.00	0.12	0.05	1.00	0.15	0.07	0.00
ß-Phellandrene	1.00	17.64	25.50	50.13	89.25	76.71	0.18	0.00	0.00	∞	∞	∞
2,2-bis(methylthio)propane	1.00	35.19	55.73	72.65	106.22	87.20	0.00	0.00	1.00	85.76	0.00	0.00
4-hexylbenzene-1,3-diyl)bis(oxy)]bis(trimethylsilane)	1.00	0.82	0.92	0.93	0.70	1.03	0.00	0.00	1.00	0.92	0.00	0.00
5-methyl-5-propylnonane	0.00	∞	∞	∞	∞	∞	0.00	0.00	1.00	104.75	1.51	1.71
1-methylethyl 2-propenyl disulfide	1.00	18.35	40.38	45.86	63.37	54.23	2.38	1.31	1.00	14.89	0.76	0.00
(E)-1-propenyl allyl disulfide	0.00	∞	∞	∞	∞	∞	0.00	0.00	1.00	14.77	0.00	0.00
Isopropyl disulfide	1.00	3.10	0.86	0.17	13.06	13.00	0.00	0.00	1.00	13.73	0.00	0.00
2-methylundecane	1.00	0.61	0.71	0.71	0.64	0.52	0.08	0.03	1.00	0.53	0.10	0.02
2,6,11-trimethyldodecane	1.00	0.38	0.93	1.04	0.97	0.70	0.20	0.07	1.00	0.04	0.30	0.00
Hexadecane	1.00	5.13	0.59	18.79	1.93	14.11	0.20	0.20	1.00	8.57	0.31	0.12
2,6-dimethylundecane	1.00	0.72	0.90	0.92	0.73	0.74	0.09	0.03	1.00	0.61	0.11	0.02
4-methyldodecane	1.00	1.14	0.72	0.81	0.78	0.53	0.09	0.03	1.00	0.65	0.16	0.02

4-ethylphenol	1.00	0.00	3.27	4.79	5.08	3.77	0.00	0.85	0.00	∞	0.00	0.00
Pentadecane	1.00	0.61	0.34	0.34	0.81	0.09	0.00	0.00	1.00	0.11	0.00	0.02
Hexylbenzene	0.00	∞	∞	∞	∞	∞	∞	0.00	0.00	0.00	∞	∞
4-methyltridecane	1.00	0.38	0.54	0.60	0.80	0.26	0.00	0.00	1.00	0.45	0.00	0.02
2-methyltridecane	1.00	0.41	0.78	0.84	0.92	0.51	0.03	0.03	1.00	0.72	0.04	0.03
dipropyl trisulfide	1.00	95.47	147.95	498.13	434.14	493.36	5.68	14.44	0.00	∞	∞	∞
3-Isopropoxy-1,1,1,7,7,7-hexamethyl-3,5,5-tris(trimethylsiloxy)tetrasiloxane	0.00	∞	∞	∞	∞	∞	∞	∞	0.00	0.00	0.00	∞
2-Tridecanone	1.00	12.96	102.08	116.38	150.96	93.42	0.00	1.65	1.00	0.00	0.00	0.00
1,14-Tetradecanediol	0.00	∞	∞	∞	∞	∞	∞	∞	0.00	0.00	∞	∞

∞ - lack of this compound in volatile profile of healthy control sample and simultaneously significant abundance in volatile profile of infected samples

Table S2. A list of VOCs detected in samples with healthy control and FOC infected bulbs in the second experiment and their relative abundance.

Chemicals/ Relative abundance	Retention time (min)	RI	Control samples			FOC infected samples		
			Brown onion	Red onion	Shallot	Brown onion	Red onion	Shallot
Argon	0.28	n.d.	1.00	1.00	1.00	0.99	1.10	1.04
2-methyl-1-propene	1.16	n.d.	1.00	1.00	1.00	0.53	2.22	0.00
Methanethiol	1.29	n.d.	0.00	0.00	0.00	∞	∞	∞
Pentane	1.52	n.d.	0.00	1.00	1.00	∞	83.37	23.64
Dimethyl ether	1.52	n.d.	1.00	1.00	1.00	0.00	0.00	0.00
Acetone	1.71	n.d.	1.00	1.00	1.00	0.19	0.42	0.43
Methyl acetate	1.86	n.d.	1.00	1.00	1.00	0.00	0.55	0.00
1-Penten-3-one	2.28	n.d.	1.00	1.00	1.00	0.00	0.00	0.00
Ethyl acetate	2.54	n.d.	1.00	1.00	1.00	0.00	0.31	0.00
Tetrahydrofuran	2.67	n.d.	1.00	1.00	1.00	0.00	1.27	0.83
Acetic acid	2.94	n.d.	1.00	1.00	1.00	0.32	0.01	0.54
Heptane	3.15	n.d.	1.00	1.00	1.00	1.76	1.35	1.29
Allyl methyl sulfide	3.32	n.d.	1.00	1.00	0.00	14.79	11.75	∞
2-Pentanone	3.40	n.d.	1.00	1.00	1.00	0.00	0.00	0.00
Toluene	4.07	747	1.00	1.00	1.00	0.05	1.23	1.25
Hexamethyl cyclotrisiloxane	4.31	770	1.00	1.00	1.00	0.48	0.76	1.80
Dibromochloro methane	4.56	794	1.00	1.00	1.00	0.67	2.18	3.68
3-methyl octane	4.92	829	1.00	1.00	1.00	2.54	1.78	2.44
3-ethyl-3-methyl- pentane	5.35	872	1.00	1.00	1.00	0.19	0.00	0.00
2.4-dimethyl- thiophene	5.35	872	1.00	1.00	1.00	0.19	0.00	0.00
1-ethyl-2-methyl benzene	5.53	890	1.00	1.00	1.00	3.22	1.36	8.59
trans-1.1.3.5-tetramethylcyclohexane	5.64	902	1.00	1.00	1.00	0.05	1.75	0.47
3-methyl nonane	5.71	909	1.00	1.00	0.00	0.00	0.39	∞
Propyl benzene	5.87	925	0.00	1.00	1.00	0.00	0.71	0.00
Dimethylphosphinic fluoride	5.94	933	1.00	1.00	1.00	0.06	1.10	1.15
Undecane	6.00	939	1.00	1.00	1.00	0.00	0.00	0.00
Octanal	6.25	966	1.00	1.00	1.00	3.78	3.68	3.00
D-Limonene	6.49	992	1.00	1.00	1.00	0.00	0.33	0.16
Dodecane	6.56	998	1.00	1.00	1.00	11.71	1.98	1.20
Nonanal	7.14	1064	1.00	1.00	1.00	0.23	0.20	0.10
Dipropyl disulfide	7.37	1091	1.00	1.00	1.00	0.64	0.63	0.18
1.2-Dithiolane	7.39	1093	1.00	1.00	1.00	3.89	2.30	5.11
Bis(n-propylthio)methane	7.47	1102	1.00	1.00	1.00	2.60	2.47	13.92
3.3-dimethyl hexane	8.50	1229	0.00	0.00	0.00	∞	∞	∞
Methenamine	8.66	1250	1.00	1.00	1.00	0.00	0.00	0.78
2-Undecanone	8.67	1251	1.00	1.00	1.00	0.00	0.00	1.34
2-hexyl-5-methyl-3(2H)-furanone	8.87	1277	1.00	1.00	1.00	9.77	1.47	6.55
6.6-Dimethyl-10-methylene-1-oxa-spiro[4.5]decane	10.08	1445	1.00	1.00	1.00	19.03	1.83	3.61
Cetene	10.67	1533	0.00	0.00	0.00	∞	∞	∞
bis(trimethylsilyl) mercaptoacetic acid	11.40	1648	1.00	0.00	1.00	0.17	0.00	0.44

n.d. – no data,

∞ - lack of this compound in volatile profile of healthy control sample and simultaneously significant abundance in volatile profile of infected samples
